# Raman Imaging—A Valuable Tool for Tracking Fatty Acid Metabolism—Normal and Cancer Human Colon Single-Cell Study

**DOI:** 10.3390/ijms25084508

**Published:** 2024-04-19

**Authors:** Karolina Beton-Mysur, Monika Kopec, Beata Brozek-Pluska

**Affiliations:** Laboratory of Laser Molecular Spectroscopy, Institute of Applied Radiation Chemistry, Faculty of Chemistry, Lodz University of Technology, Wroblewskiego 15, 93-590 Lodz, Poland; karolina.beton@dokt.p.lodz.pl (K.B.-M.); monika.kopec@p.lodz.pl (M.K.)

**Keywords:** Raman spectroscopy, Raman imaging, fatty acids, colon cancer, metabolism, cancer biomarkers

## Abstract

Altered metabolism of lipids is a key factor in many diseases including cancer. Therefore, investigations into the impact of unsaturated and saturated fatty acids (FAs) on human body homeostasis are crucial for understanding the development of lifestyle diseases. In this paper, we focus on the impact of palmitic (PA), linoleic (LA), and eicosapentaenoic (EPA) acids on human colon normal (CCD-18 Co) and cancer (Caco-2) single cells using Raman imaging and spectroscopy. The label-free nature of Raman imaging allowed us to evaluate FAs dynamics without modifying endogenous cellular metabolism. Thanks to the ability of Raman imaging to visualize single-cell substructures, we have analyzed the changes in chemical composition of endoplasmic reticulum (ER), mitochondria, lipid droplets (LDs), and nucleus upon FA supplementation. Analysis of Raman band intensity ratios typical for lipids, proteins, and nucleic acids (I_1656_/I_1444_, I_1444_/I_1256_, I_1444_/I_750_, I_1304_/I_1256_) proved that, using Raman mapping, we can observe the metabolic pathways of FAs in ER, which is responsible for the uptake of exogenous FAs, de novo synthesis, elongation, and desaturation of FAs, in mitochondria responsible for energy production via FA oxidation, in LDs specialized in cellular fat storage, and in the nucleus, where FAs are transported via fatty-acid-binding proteins, biomarkers of human colon cancerogenesis. Analysis for membranes showed that the uptake of FAs effectively changed the chemical composition of this organelle, and the strongest effect was noticed for LA. The spectroscopy studies have been completed using XTT tests, which showed that the addition of LA or EPA for Caco-2 cells decreases their viability with a stronger effect observed for LA and the opposite effect observed for PA. For normal cells, CCD-18 Co supplementation using LA or EPA stimulated cells for growing, while PA had the opposite impact.

## 1. Introduction

Altered lipid metabolism is a key indicator of many diseases including cancer [1,2]. Therefore, this class of compounds is attracting growing interest as biomarkers in clinical applications, highlighting the role of lipidomics in cancer studies.

In this paper, we focus on lipid metabolism with special emphasis on fatty acids (FAs)—saturated (palmitic acid (PA, 16:0)) and unsaturated (linoleic acid (LA, 18:2) and eicosapentaenoic acid (EPA, 20:5))—and their potential in diagnostics and therapies of colorectal cancer (CRC).

Most often, CRCs are adenocarcinomas arising from pathological changes in the mucosa’s epithelial cells [3]. Approximately 30% of CRCs are associated with hereditary gene mutations. Disfunctions of repair genes are responsible for around 15% of CRCs; the other 80–85% is associated with mutations in adenomatous polyposis coli gene (APC). Furthermore, CRCs may develop as a consequence of inflammatory bowel disease (IBD) [4]. Two of the most common genetic defects found in CRCs are KRAS and p53 mutations. Moreover, it has been shown that these mutations are associated with enhanced proliferation of cancerous cells [5,6].

Evidence of lipid reprogramming metabolism in cancer cells was first reported in the 1920s, when the Warburg effect was described the first time [7]. However, nowadays, a shift towards the reversed Warburg effect is popular, since researchers proved that each type of cancer cell has unique metabolic features and some may synthesize ATP by means of oxidative phosphorylation [8]. The metabolic pathways of lipids that have been affected in CRC cells include, among others, the synthesis, desaturation, elongation, and mitochondrial oxidation of FAs.

Generally, lipids can be described as a diverse group of compounds. LIPID MAPS [9] classified them (based on the presence of isoprene and ketoacyl groups) as follows: FAs, sphingolipids, sterol lipids, glycerolipids, glycerophospholipids, prenol lipids, saccharolipids, and polyketides [10].

However, for the proper functioning of organisms, polyunsaturated fatty acids (PUFAs) are required. These acids are formed from palmitic acid (PA) as a result of the action of desaturases causing the introduction of a double bond into the structure of the acid molecule. From PA, palmitoleic acid (C16:1 ω-7) is formed, and from stearic acid, and oleic acid (OA, C18:1 ω-9) is formed. As a result of the action of Δ12-desaturase, oleic acid is converted to linoleic acid (LA, C18:2 ω-6), which is further converted by Δ15-desaturase to α–linolenic acid (ALA, C18:3 ω-3).

However, in animal tissues, double bonds can only be introduced between the already existing double bond and the carboxyl group due to the lack of suitable desaturases. Therefore, the synthesis of LA and ALA does not take place in animal tissues, and they must be supplied in the diet [11].

The detailed description of FA biosynthesis and schematic presentation of FA biosynthesis is shown in Figure S1 provided in the Supplementary Materials (SM). Please see Figure S1 in the Supplementary Materials.

**Figure 1:**
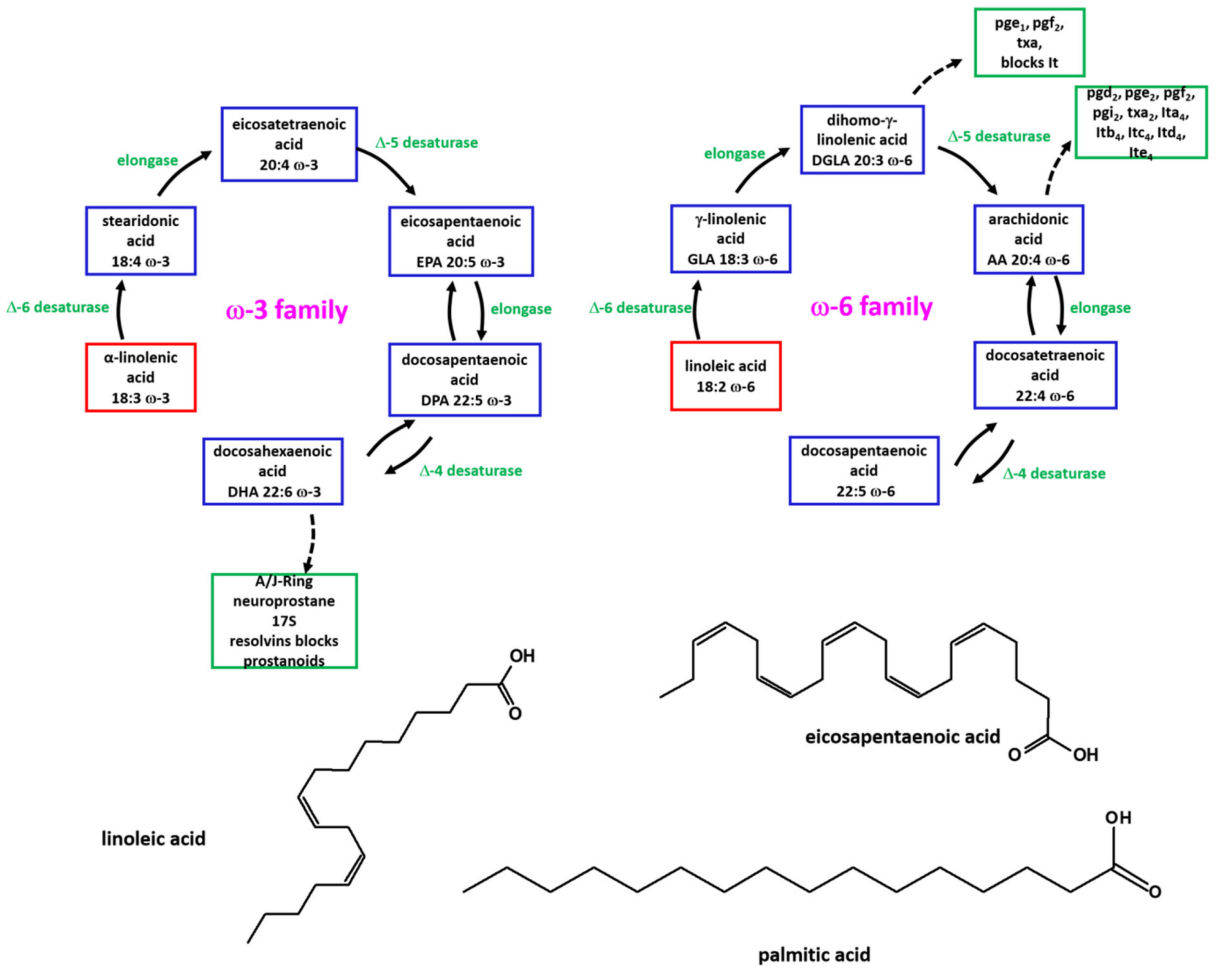
Schematic representation of formation of eicosanoids by ω-3 and ω-6 FAs.

Figure 1 shows the correlation between the structure of FAs and formation of eicosanoids.

PA is a fatty acid with a 16-carbon chain. It is the most common saturated FA found in animals, plants, and microorganisms. In the human body, PA may participate in the regulation of hormone secretion as well as in the transmission of signals between body cells. PA may also support the proper functioning of the immune system [12]. However, it must be emphasized that the positive effect of this compound can be observed only for small doses of PA [13,14,15]. Contrarily, a high consumption of PA in the daily diet may have a negative impact on the human body, contributing to an increase in the concentration of total cholesterol in the blood serum and an increase in its LDL (low-density lipoprotein) fraction [16,17,18,19]. This, in turn, may increase the risk of atherosclerosis and cardiovascular diseases. Additionally, excessive consumption of saturated fatty acids (SFAs), including PA, may increase the risk of obesity and digestive disorders [20,21,22]. Many research groups have also proven the correlation between PA overconsumption and cancer development [23]. Through lipidomics analysis, Lin et al. demonstrated that PA can impact the aggressiveness of cancer cells. The authors highlighted the changes in cell membrane fluidity and glucose metabolism regulation [24]. Sun et al. demonstrated that PA may regulate the expression of genes involved in the metabolism of FAs, such as fatty acid synthase gene (FASN), stearoyl-CoA desaturase-1 (SCD1), and elongation of long-chain FA family member 6 (ELOVL6), which are directly associated with gastric cancer [25]. Zhang et al. investigated the impact of PA on the genes pyruvate dehydrogenase kinase 4 (PDK4) and ACSL5, which stimulate the proliferation of lung cancer cells [26]. Metastasis of cancer cells upon FA supplementation was also investigated by Pascual et al. [27]. Researchers conducted experiments on human oral squamous cell carcinoma (OSCC). They supplemented OSCC with PA, OA, and LA before injecting them into mice. The study revealed that pretreatment with PA, in contrast to OA or LA, significantly increased the formation of metastases. Notably, cells treated with PA showed metastatic properties even after PA removal for 2 weeks or treatment with OA.

Therefore, nutrition experts and medical doctors recommend replacing SFAs with essential unsaturated fatty acids (UFAs), the consumption of which can have beneficial effect on human health and positively affect the functioning of the body [28,29,30,31].

LA is a fatty acid with an 18-carbon chain, 2 double bonds and typically occurs as a triglyceride in nature rather than as a free FA. LA is one of two essential FAs for humans, and serves also as a precursor to arachidonic acid, which is a parent molecule for prostaglandins, leukotrienes, thromboxane, endocannabinoids and eicosanoids [32]. Large scale human epidemiological studies indicated that high intakes of LA protect against cancer development [33]. The first beneficial effect of CLA (conjugated LA) as anti-cancer agent was discovered by M. Pariza [34]. Synthetically prepared CLA isomers were applied by researchers to mice prior to the initiation of epidermal carcinomas. Mice that received CLAs developed only half the number of papillomas [35]. Subsequent studies have proved also that other murine carcinoma models show an improvement with CLA supplementation, including mammary [36], colon [37], stomach [38] and prostate cancers [39].

EPA is a fatty acid with a 16-carbon chain and 5 double bonds, and is a precursor for prostaglandin-3, thromboxane-3, and leukotriene-5 eicosanoids. EPA is also both a precursor and a hydrolytic breakdown product of eicosapentaenoyl ethanolamide [40]. Bie et al. have shown that EPA attenuates ovarian cancer by improving immunomodulation. The immunomodulatory effects of EPA were associated with PI3K/Akt, ERK1/2 and NF-κB P65 expression [41]. Moreover, Ando et al. proved that EPA suppresses angiogenesis by reducing the secretion of IL-6 and VEGF from colon-cancer-associated fibroblasts [42]. It has also been shown that EPA deceases systemic inflammation caused by IL-6 [43] and decreases inflammation caused by cancers [44].

Visualizing the presence and distribution of fatty acids within cells, both in their free and esterified forms, is crucial for comprehending how these molecules are integrated, stored, and processed. However, existing techniques for imaging multiple intracellular fatty acids have been hindered by their minute size, posing challenges in labeling and tracking without altering their essential biological and biophysical properties. Here, we introduce a novel approach for visualizing intracellular fatty acids and their accumulation within specific cell organelles. Leveraging distinct Raman spectra associated with different labeling patterns, our Raman imaging technique enables the identification and tracking of fatty acids within cells. Our findings reveal that fatty acids with a higher double bond content tend to accumulate more prominently within endoplasmic reticulum and lipid droplets. This innovative methodology not only sheds light on the spatial dynamics of fatty acids but also holds promise for elucidating the behavior of various other metabolites within cells. Research into the detection of fatty acids, their storage sites or the tracking of fat metabolism products has been successfully carried out by various research groups from around the world. Lipid metabolism and its mechanism have been the subject of research by scientists from all over the world; however, Raman spectroscopy is a unique method in this aspect as it gives very precise, unambiguous results, allowing the registration of even the smallest changes occurring in the cell as a result of fatty acid supplementation [45,46,47,48,49,50,51,52,53].

**Figure 2:**
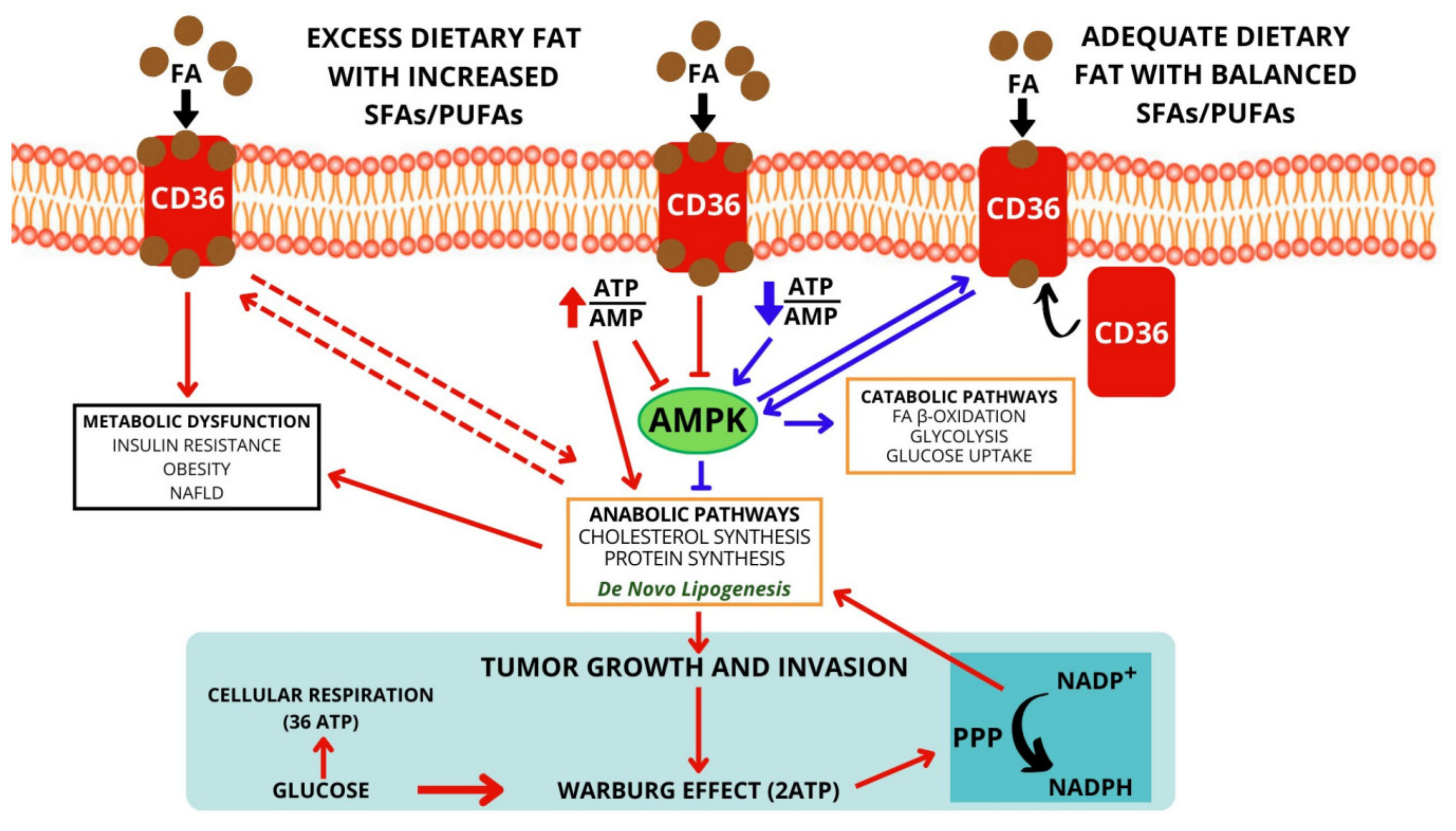
Metabolic pathways in normal and excess dietary fat with increased or balanced SFA/PUFA ratio.

In summary, an increased incidence of cancers (including CRCs) is typical among people consuming a larger amount of animal saturated fats, with a diet rich in myristic, lauric, and palmitic acids. In contrast, a reduction in morbidity under the influence of ALA, DHA, and EPA was observed in relation to stomach [54], pancreas [55,56], colon [57,58], lung [59], breast [60,61], and prostate cancer [62]. Figure 2 shows the simplified metabolic pathways in normal and excess dietary fat with an increased or balanced SFA/PUFA ratio.

All of the aforementioned factors confirm the crucial role of FAs in human body homeostasis and provide compelling topics for new investigations. In this paper, we focus on the impact of PA, LA, and EPA on human colon single cells using Raman imaging and spectroscopy. The spectroscopy studies were completed with XTT tests analyzing the viability of normal CCD-18 Co and cancer Caco-2 human colon cells upon FA supplementation.

## 2. Results

The uncontrolled cellular growth typical of cancer development requires a constant supply of nutrients. The most crucial role in an unbalanced diet stimulating tumors growth is played by sugars and fats. Since the 1970s, numerous studies proved the adverse impact of sugars and fats on human health including increased cancer risk [63]. There is convincing evidence that excess body weight is associated with an increased risk for many cancers including endometrial, esophageal, hepatocellular carcinoma; renal and pancreatic adenocarcinomas; gastric cardia cancer; meningioma; multiple myeloma; and colorectal, postmenopausal breast, ovarian, gallbladder and thyroid cancers [64].

To identify the impact of FAs on human colon cells, we established a single-cell analysis approach, which, in the first step, couples the analysis of Raman maps and mean single Raman spectra. To properly address biochemical changes, with the main focus on the metabolism of FAs in human normal and cancer colon cells upon FA supplementation in comparison to not supplemented types, we systematically investigated how the Raman method responds to in vitro samples. We used spontaneous Raman spectroscopy to record vibrational spectra and Raman maps at the single-cell level for human colon cell lines: CCD-18 Co (normal) and Caco-2 (cancer) over the molecular spectral range of 500–1800 cm^−1^ (the fingerprint region).

**Figure 3:**
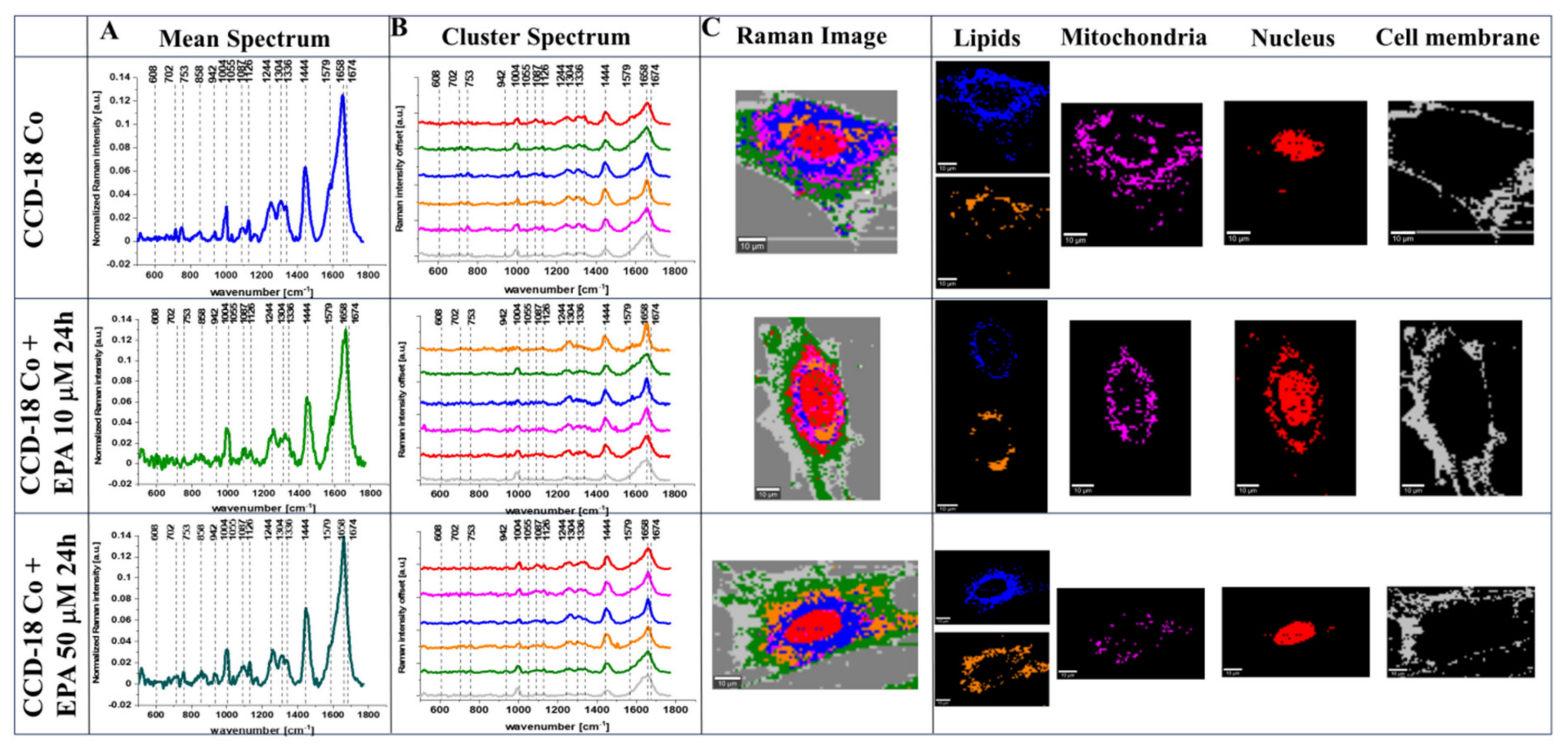
The mean Raman spectra for cells as a whole (**A**), the mean Raman spectra typical for all clusters identified by using cluster analysis (CA) (**B**), Raman images constructed based on CA (**C**), and the single clusters wherein blue and orange correspond to lipid rich regions, magenta corresponds to mitochondria, red corresponds to nucleus, and gray corresponds to cell membrane identified by using CA for human normal colon cells CCD-18 Co without any supplementation and upon EPA supplementation for 24 h, for 10 μM and 50 μM. All cells were measured in PBS. The scale bar represents 10 μm. Colors of the spectra correspond to the colors of clusters.

**Figure 4:**
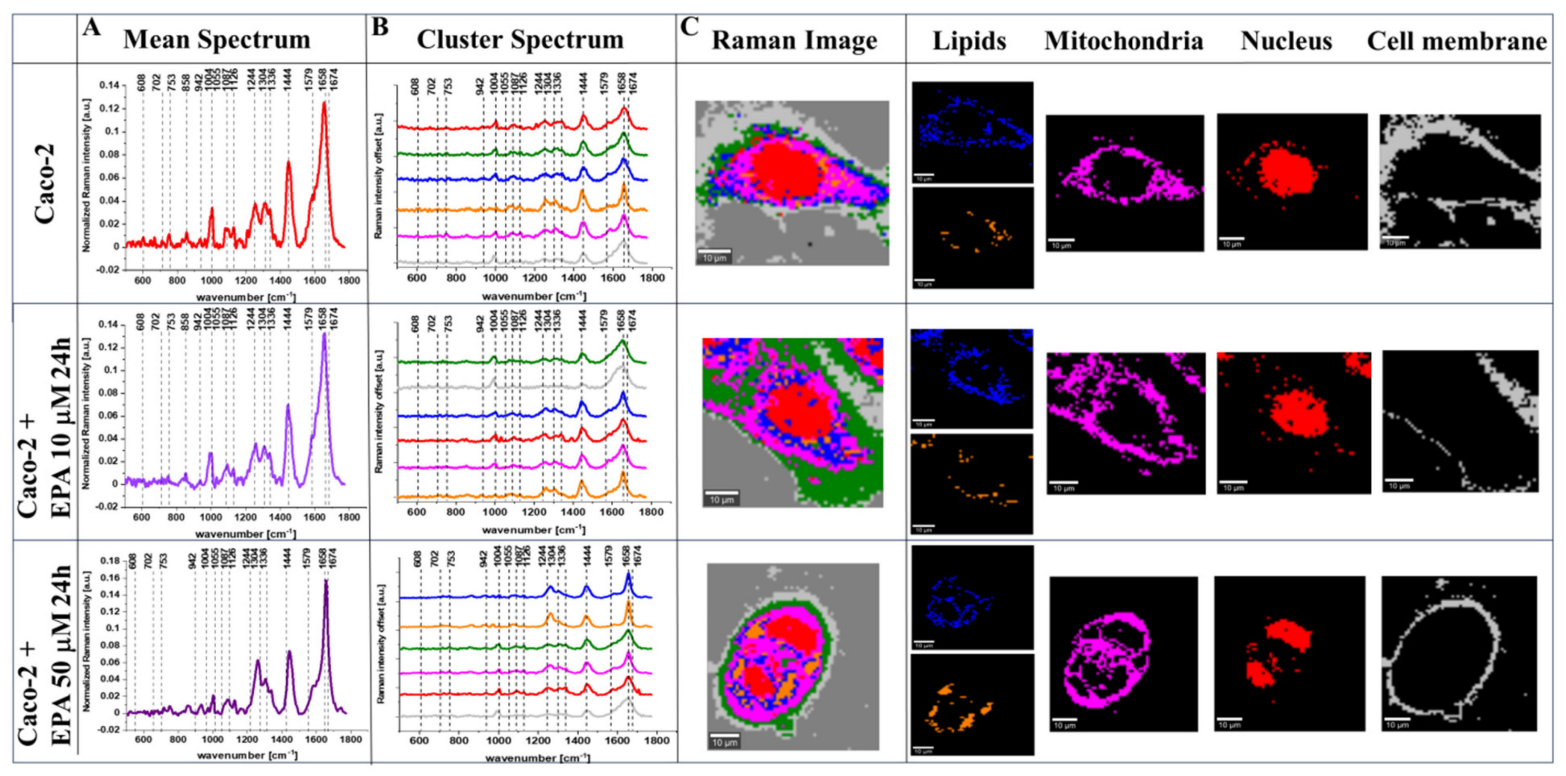
The mean Raman spectra for cells as a whole (**A**), the mean Raman spectra typical for all clusters identified by using cluster analysis (CA) (**B**), Raman images constructed based on CA (**C**), and the single clusters wherein blue and orange corresponds to lipid rich regions, magenta corresponds to mitochondria, red corresponds to nucleus, and gray corresponds to cell membrane identified by using CA for human colon cancer cells Caco-2 without any supplementation and upon EPA supplementation for 24 h, for 10 μM and 50 μM. All cells were measured in PBS. The scale bar represents 10 μm. Colors of the spectra correspond to the colors of clusters.

Figure 3 shows the Raman data obtained for human colon normal cells (CCD-18 Co) including cells upon EPA supplementation (for transparency of data presentation, we show the data for one cell). The data for Caco-2 human colon cancer cells including the EPA-supplemented type are presented in Figure 4. Data for LA supplementation are presented in Figure S3 (for CCD-18 Co line) and Figure S4 (for Caco-2 line), and data for PA supplementation are shown in Figure S5 (for CCD-18 Co line). Each figure contains panels presenting intensity spectra with an interpretation of individual components and assignment to individual cellular organelles, which we explain in the Section 3.

The results for LA and PA are presented in Figures S3–S5 in the Supplementary Materials.

One can see from Figure 3, Figure 4 and Figures S3–S5 that Raman imaging (RI) can be used to obtain detailed information regarding the subcellular structure of each type of human colon cell: normal, cancer, and normal or cancer upon FA supplementation. Based on CA, which is a well-established method for RI data elaboration [65], we have identified, for each cell: the endoplasmic reticulum (ER, blue), the lipid droplets (LDs, orange), the cytoplasm (green), the nucleus (red), the mitochondria (magenta), and the cell membrane (gray). Moreover, Raman spectroscopy allowed us to obtain well-resolved vibrational spectra based on which one can identify the main chemical compounds: nucleic acids, lipids, proteins, saccharides, etc.

The usefulness of RI for single-cell analysis at the level of individual organelles has been proven in our previous papers [66,67,68,69]. Moreover, fluorescence staining confirmed the effectiveness of RI for individual structure visualization and correctness of Raman data interpretation [70,71,72,73,74,75]. Scheme S1 in the Supplementary Materials shows the simplified illustration of the RI experiment idea and the comparison of RI and fluorescence data for one cell chosen from our database.

Lipidomics studies are so important because altered lipid metabolism has been observed for drug resistance; e.g., increased de novo lipogenesis mediated by FAS facilitated gemcitabine resistance in pancreatic cancer [76]. Moreover, it has been shown that the cancer-associated adipose tissue promoted resistance to anti-angiogenic factors by supplying FAs to cancer cells in regions where the glucose demand was insufficient [77]. Additionally, it has been shown that LD production mediated by lysophosphatidylcholine acyltransferase2 increased the resistance of CRC cells to 5-fluorouracil and oxaliplatin [78].

## 3. Discussion

All facts mentioned above justify the additional studies on the metabolism of FAs at the subcellular level.

**Scheme 1:**
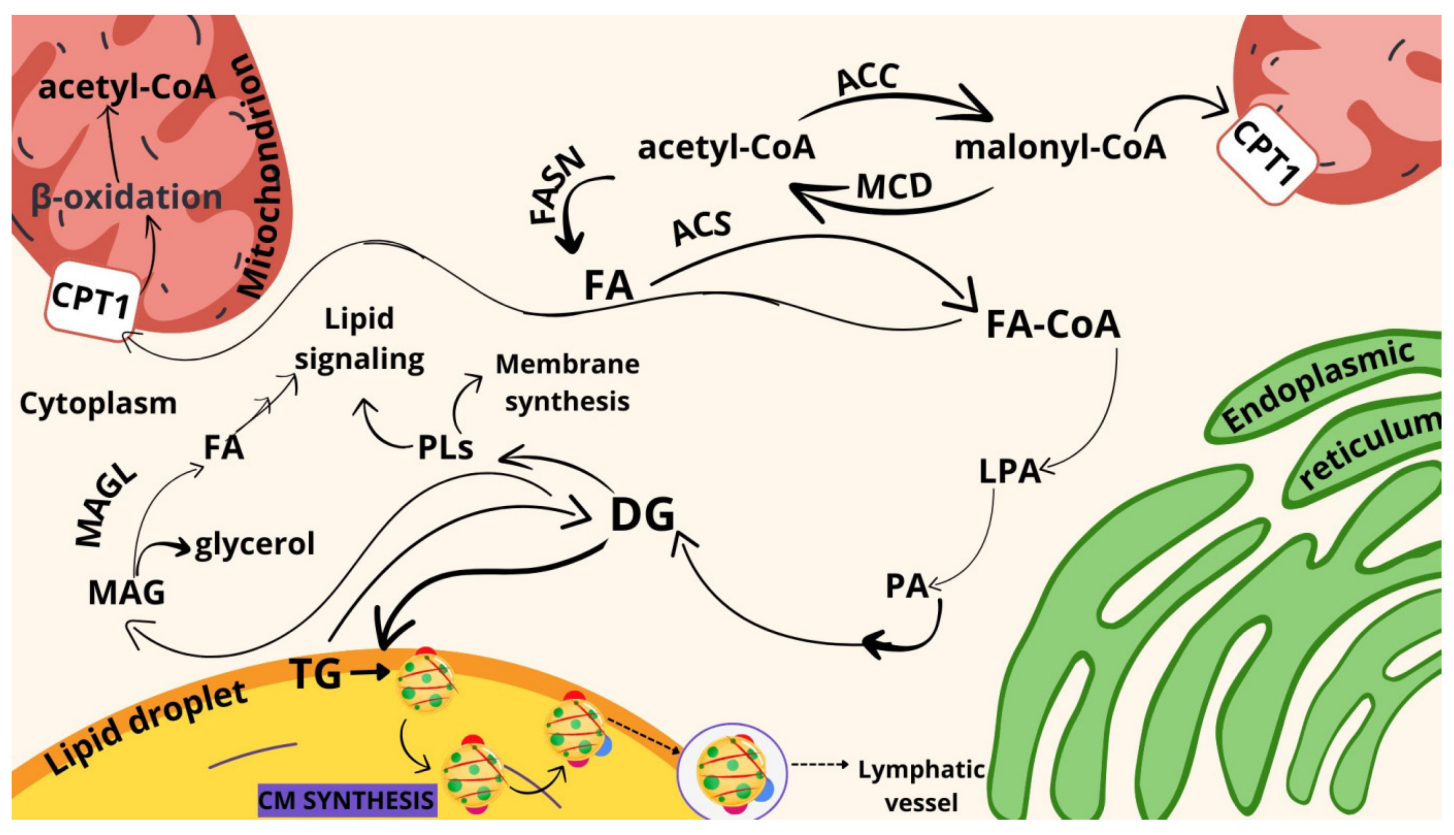
Schematic representation of FA synthesis and metabolism in human colon cells. Explanation of the abbreviations from scheme: **DG:** diacylglycerol, **FA**: fatty acid, **LPA:** lysophosphatidic acid, **MAG:** monoacylglycerol, **TG:** triacylglycerol, **ACC:** acetyl-CoA carboxylase, **ACS:** acetyl-CoA synthetase, **FASN:** fatty acid synthase, **FA-CoA:** fatty acid-coenzyme A, **MAGL:** monoacylglycerol lipase, **MCD:** malonyl-CoA decarboxylase, **CPT1:** carnitine palmitoyltransferase I, **PA:** phosphatidic acid, **PLs:** polar lipids.

When analyzing the pathways of the metabolism of FAs in the human colon, one has to remember that FAs gain entrance into the intestine through enterocytes, which are placed on the inner surface of the colon. FAs and monoglycerides (MGs) are able to cross the apical membrane of the intestinal absorptive cells via passive diffusion or high-specialized fatty acid transport protein 4 (FATP4), or CD36 may be involved in this process. Subsequently, FA molecules are bound to intestinal fatty-acid-binding proteins (I-FABP), which transport them to the ER. Scheme 1 shows the simplified pathways of dietary lipid metabolism in human colon cells.

The ER is a major hub for the metabolism of FAs, being implicated in uptake of exogenous FAs, de novo synthesis, elongation, and desaturation. The ER is in contact with many other organelles, such as mitochondria, the nucleus, LDs, and peroxisome, via their membranes, which allows efficient transfer of FA substrates and enzymes. The excess FAs present in the ER are used by cells for triacylglycerol (TAG) and cholesterol ester (ChE) synthesis. Subsequently, TAGs and ChEs are stored in LDs or exported through the organism by lipoproteins (many FAs act as specific platforms with high protein affinity and are used as a substrate for protein acylation, affecting their activity and localization). The primary lipoproteins synthesized at the ER in enterocytes are the chylomicrons (CM), also known as ultra-low-density lipoproteins (ULDLs). ULDLs enable fats to move within the water-based solution of the bloodstream (ApoB48 is a protein specific to CM).

**Figure 5:**
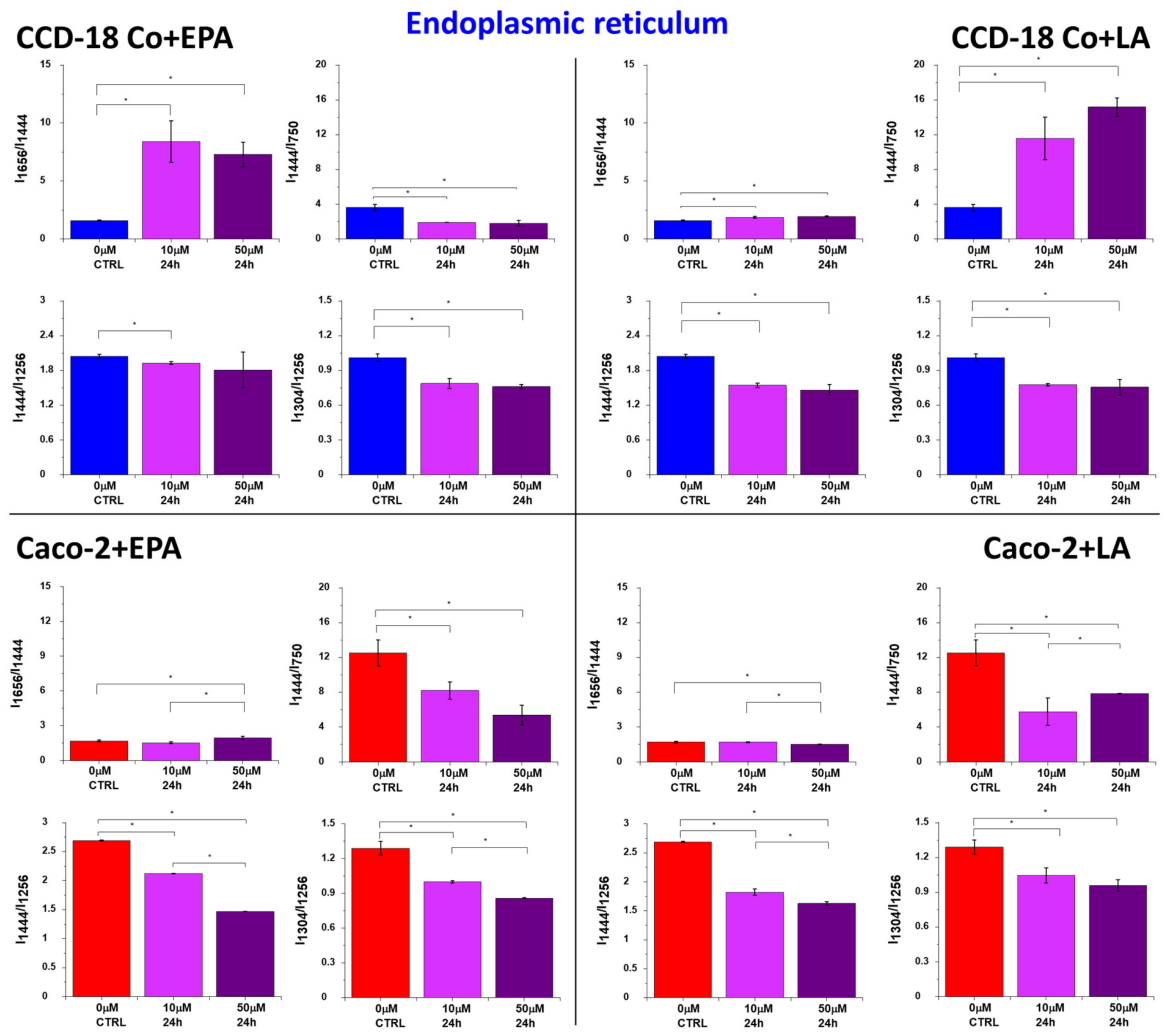
Raman band intensities ratios based on peaks typical for lipids (1304, 1444 cm^−1^), proteins/nucleic acids (750 cm^−1^), and proteins (1256, 1656 cm^−1^) calculated based on the mean Raman spectra obtained for the ER (blue cluster in RI data). Data are presented as mean ± SD. The statistically significant results, based on ANOVA analysis, have been marked with asterisks (confidence level = 0.05).

**Figure 6:**
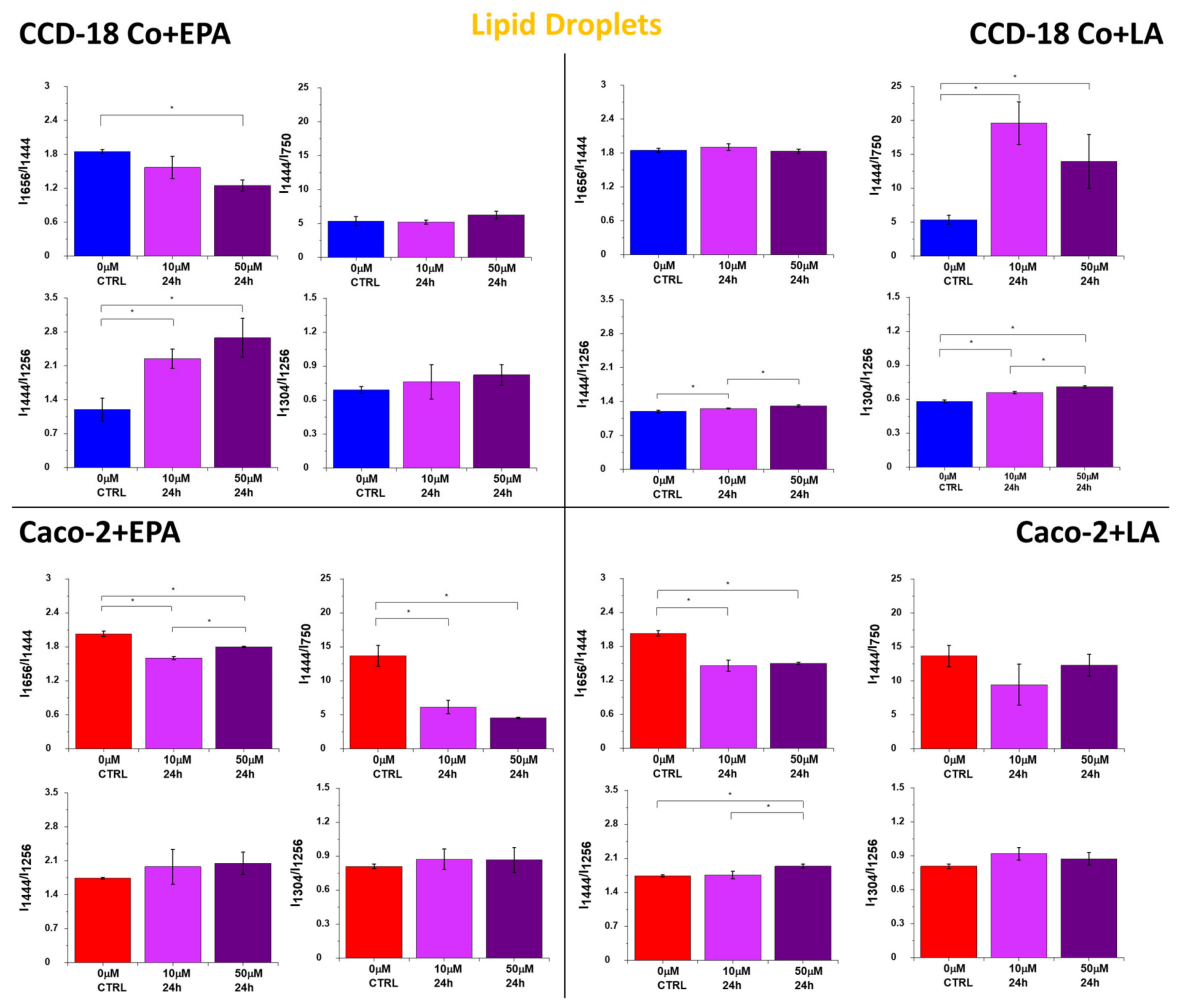
Raman band intensity ratios based on peaks typical for lipids (1304, 1444 cm^−1^), proteins/nucleic acids (750 cm^−1^), and proteins (1256, 1656 cm^−1^) calculated based on the mean Raman spectra obtained for LDs (orange cluster in RI data). Data are presented as mean ± SD. The statistically significant results, based on ANOVA analysis, have been marked with asterisks (confidence level = 0.05).

**Figure 7:**
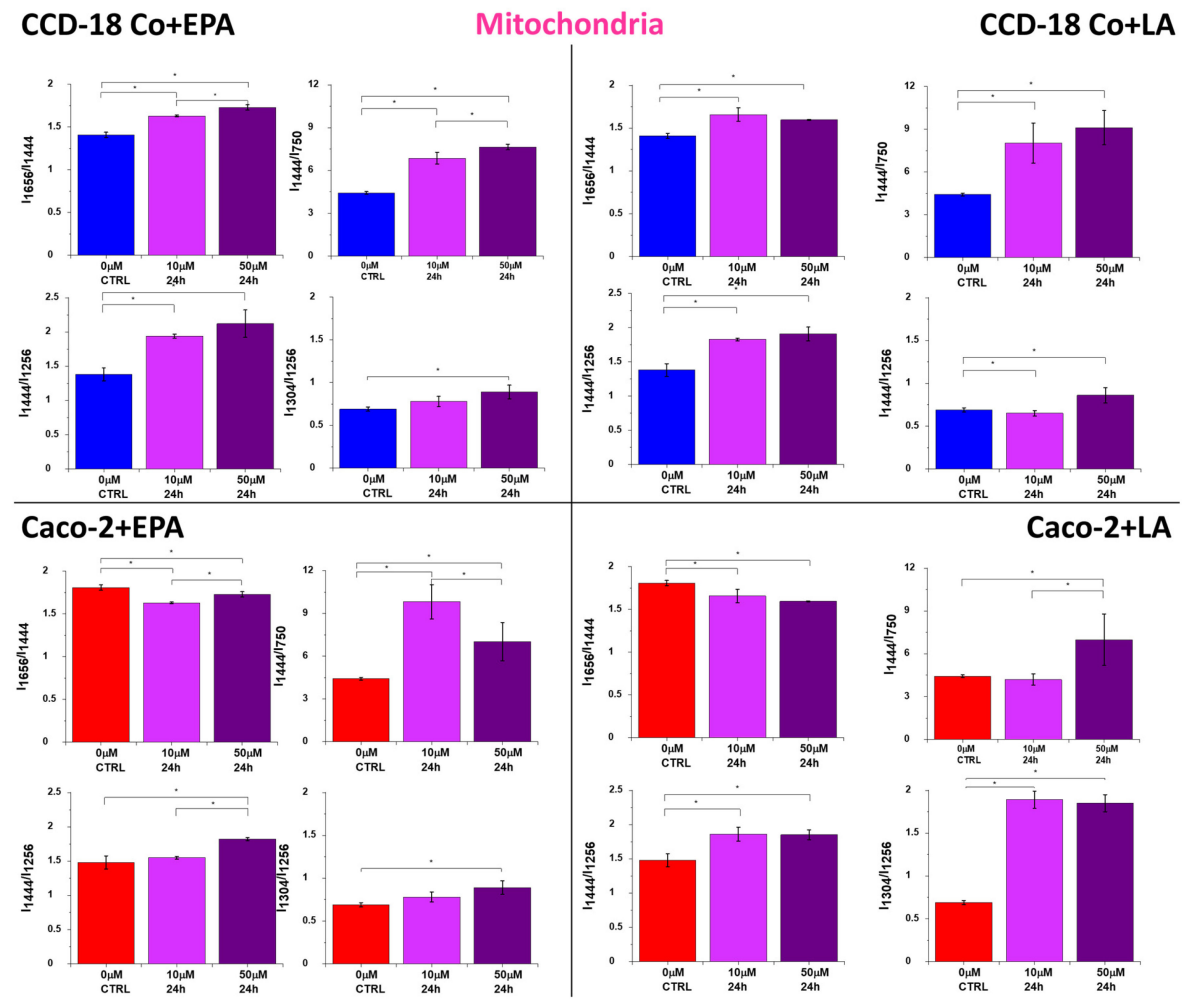
Raman band intensity ratios based on peaks typical for lipids (1304, 1444 cm^−1^), nucleic acids (750 cm^−1^), and proteins (1256, 1656 cm^−1^) calculated based on the mean Raman spectra obtained for mitochondria (magenta cluster in RI data). Data are presented as mean ± SD. The statistically significant results, based on ANOVA analysis, have been marked with asterisks (confidence level = 0.05).

**Figure 8:**
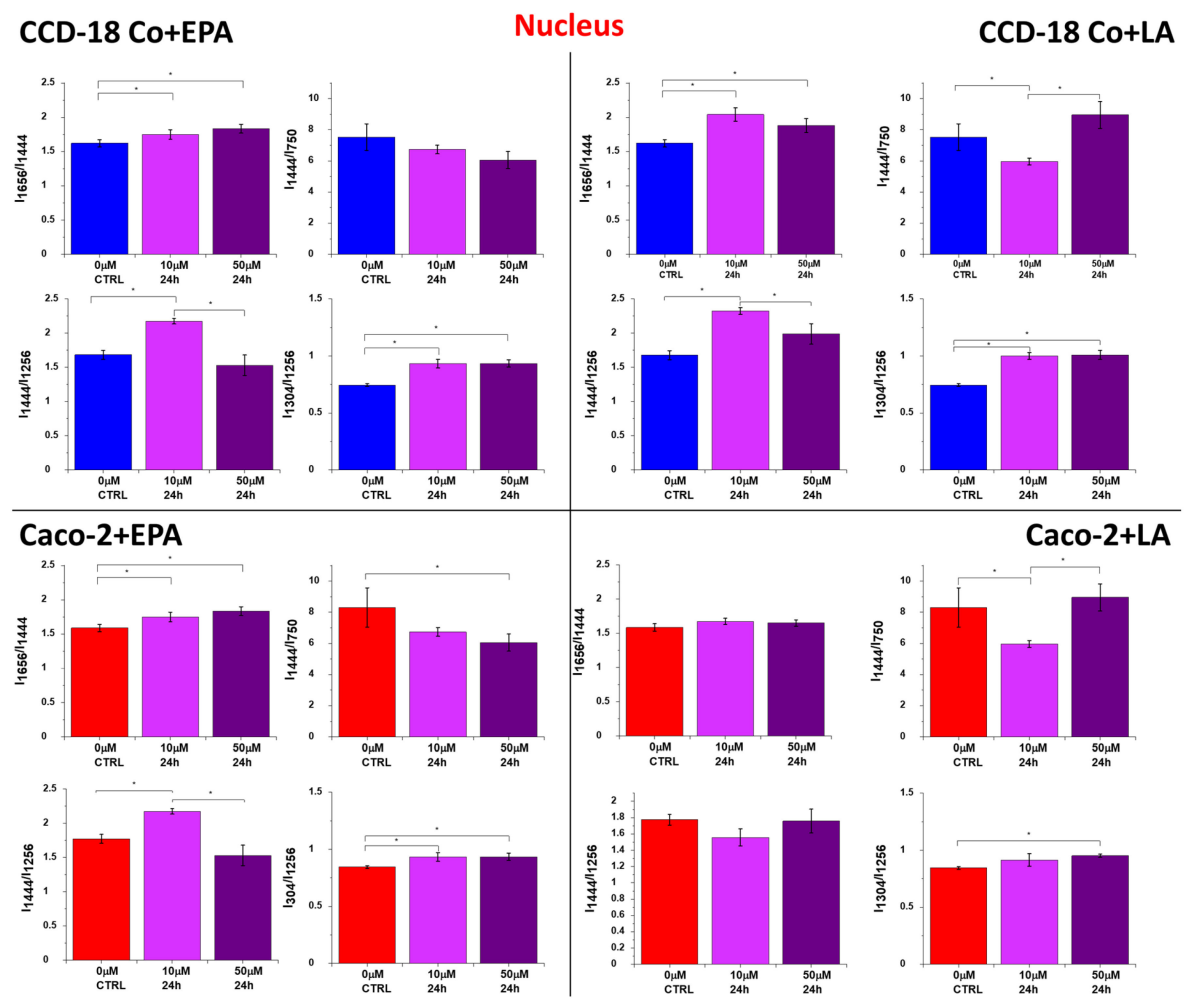
Raman band intensity ratios based on peaks typical for lipids (1304, 1444 cm^−1^), nucleic acids (750 cm^−1^), and proteins (1256, 1656 cm^−1^) calculated based on the mean Raman spectra obtained for the nucleus (red cluster in Raman imaging data). Data are presented as means value ± SD. The statistically significant results, based on ANOVA analysis, have been marked with asterisks (confidence level = 0.05).

**Figure 9:**
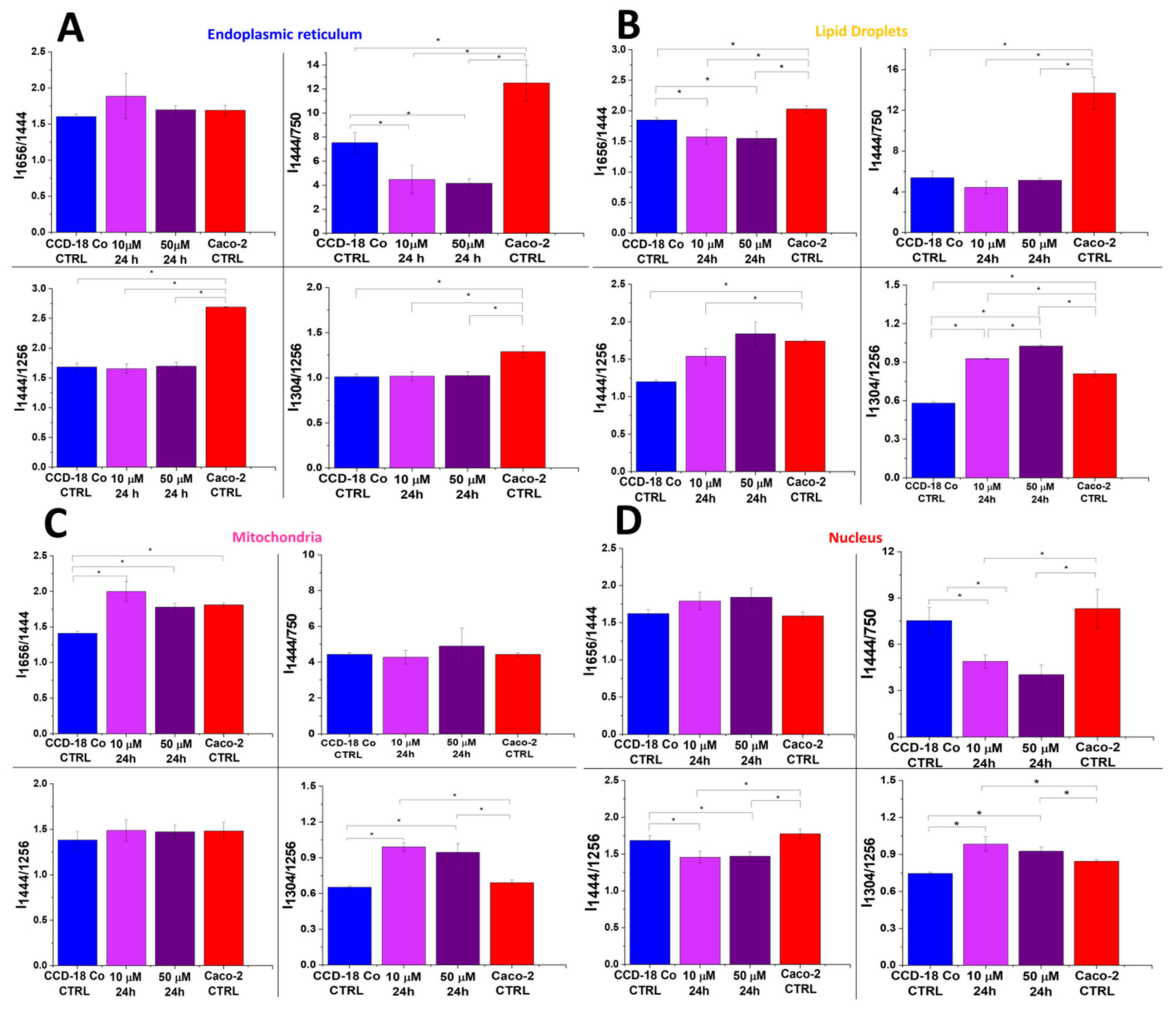
Raman band intensity ratios based on peaks typical for lipids (1304, 1444 cm^−1^), proteins/nucleic acids (750 cm^−1^), and proteins (1256, 1656 cm^−1^) calculated based on the mean Raman spectra obtained for ER (panel (**A**), blue cluster in RI data), LDs (panel (**B**), orange cluster in RI data), mitochondria (panel (**C**), magenta cluster in RI data), and nucleus (panel (**D**), red cluster in RI data). Data are presented as means value ± SD. The statistically significant results, based on ANOVA analysis, have been marked with asterisks (confidence level = 0.05).

**Figure 10:**
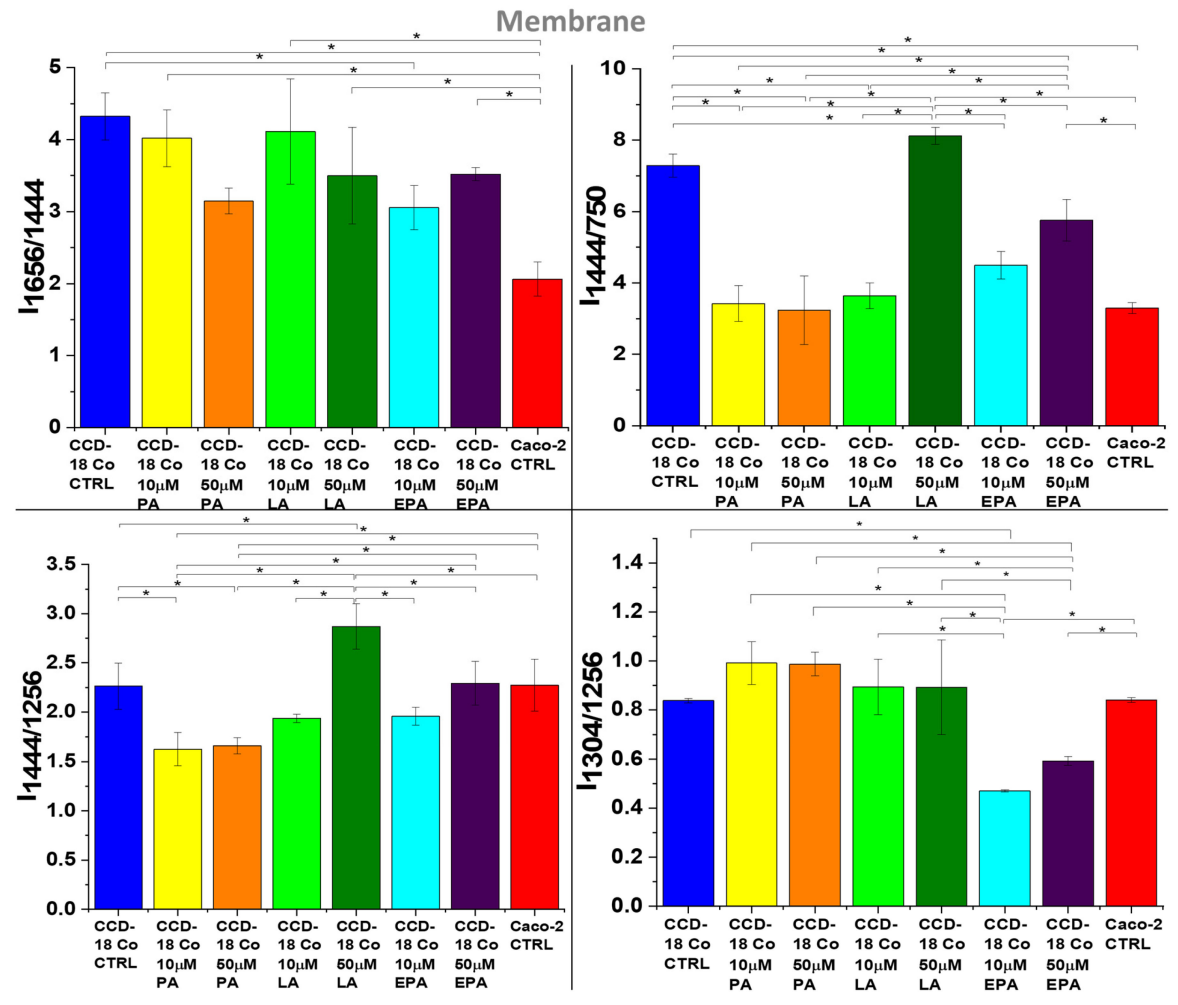
Raman band intensity ratios based on peaks typical for lipids (1304, 1444 cm^−1^), proteins/nucleic acids (750 cm^−1^), and proteins (1256, 1656 cm^−1^) calculated based on the mean Raman spectra obtained for membrane (gray cluster in RI data) for normal colon cells—CCD-18 Co (blue), CCD-18 Co upon PA supplementation (10 μM—yellow, 50 μM—orange), CCD-18 Co upon LA supplementation (10 μM—lime, 50 μM—green), CCD-18 Co upon EPA supplementation (10 μM—turquoise, 50 μM—violet), and cancer colon cells—Caco-2 (red). Data are presented as means value ± SD. The statistically significant results, based on ANOVA analysis, have been marked with asterisks (confidence level = 0.05).

Figure 3, Figure 4 and Figures S3–S5 present the analysis of cells using Raman spectroscopy and the analysis of areas corresponding to lipid-rich regions, mitochondria, the nucleus, and the cell membrane. Based on the obtained spectra and Raman images, a spectral analysis of characteristic bands was performed, and in further considerations, based on the identified bands, we analyzed the cellular metabolism of fatty acids, as shown in the Figure 5, Figure 6, Figure 7, Figure 8, Figure 9 and Figure 10 using the Raman band intensity ratios.

The increased number of lipoproteins synthesized at the ER upon FA supplementation can be observed by using Rama spectra. Figure 5 shows the ratios of Raman band intensity characteristic for proteins/nucleic acids (750 cm^−1^), proteins (1256, 1656 cm^−1^), and lipids (1304, 1444 cm^−1^) upon LA and EPA supplementation. The increasing synthesis of proteins specialized in FA transport after the addition of acids, for the ER, is confirmed by the tendency observed for I_1656_/I_1444_, I_1444_/I_1256_, I_1304_/I_1256_, and I_1444_/I_750_ ratios (decreasing for I_1656_/I_1444_ and I_1444_/I_750_ and increasing for I_1444_/I_1256_ and I_1304_/I_1256_).

Moreover, the concentration effect is observed, and all regularities are noticed for both types of human colon cells: normal CCD-18 Co and cancer Caco-2.

Lipoproteins synthetized in the ER are then moved to LDs, which are specialized in lipid storage. LDs consist of the core built by neutral lipids, mainly TGAs, ChEs, and a monolayer mainly formed by phospholipids (phosphatidylcholine, phosphatidylethanolamine, and phosphatidylinositol [79]). The surface of LDs is decorated by proteins that are specialized in lipid metabolism regulation. The first and best-characterized coat protein family is the perilipin protein family, consisting of five proteins: perilipin 1 (PLIN1), perilipin 2 (PLIN2/ADRP), perilipin 3 (PLIN3/TIP47), perilipin 4 (PLIN4/S3-12), and perilipin 5 (PLIN5/OXPAT/LSDP5/MLDP) [80,81,82]. Figure 6 shows the ratios of Raman band intensities characteristic for proteins/nucleic acids (750 cm^−1^), proteins (1256, 1656 cm^−1^), and lipids (1304, 1444 cm^−1^) in LDs.

The tendency observed for Raman peak intensity ratios in Figure 6 is the expected result for cells faced with the task of intensive transfer of excess FAs to LDs. Moreover, the concentration effect is observed, and all regularities are noticed for both types of human colon cells: normal CCD-18 Co and cancer Caco-2, even if for normal cells the stronger effect is noticed.

FAs are also used in mitochondria to produce energy. Mitochondria are responsible for energy production in the form of ATP, which is crucial for the proper functioning of cells. In mitochondria, FAs undergo β-oxidation, which generates acetyl-coenzyme-A, flavin adenine dinucleotide (FADH_2_), and nicotinamide adenine dinucleotide (NADH). Mitochondria play a crucial role in the β-oxidation of FAs as they are the site of acetyl-CoA production and the citric acid cycle. However, mitochondrial stress can be observed as a concrescence of elevated β-oxidation; increased ROS production may result in cell damage or cell death. Moreover, it should be noted that lipid overload of the mitochondria is directly connected to insulin resistance, which is crucial for type 2 diabetes during obesity. One can see from Figure 7 that all ratios combining the intensity of Raman peaks for proteins/nucleic acids (750 cm^−1^), proteins (1256, 1656 cm^−1^), and lipids (1304, 1444 cm^−1^) confirm the increasing activity of mitochondria in cancer Caco-2 cells; for the normal cells, CCD-18 Co, the correlation is opposite, even if the concentration effect is observed in both types of cells.

FAs can be also transported to the nucleus. The special role in such a transport is played by the fatty-acid-binding proteins (FABPs), which have the ability to transport FAs not only to the nucleus but also to the mitochondria and ER. FABPs also participate in the uptake of FAs from the extracellular environment. By transporting FAs to the nucleus, FABPs can modulate the activity of nuclear receptors involved in transcriptional regulation. Altered expression of certain FABPs has been observed in various cancers [83]; therefore, FABP levels may serve as cancer development biomarkers. I-FABPs have been investigated as potential biomarkers for IBD [84]. High levels of I-FABPs in serum may indicate intestinal mucosal damage [85]. One can see from Figure 8 that all ratios combining the intensity of Raman peaks for proteins (1256, 1656 cm^−1^), lipids (1304, 1444 cm^−1^), and nucleic acids (750 cm^−1^) confirm the increasing activity of lipoproteins for both types of cells in nucleus. Moreover, the dose effect for EPA and LA is observed.

As we discussed above, the excess consumption of PA, in contrast to UFAs (LA and EPA), can have a negative impact on the human body. To check the correlation between the human colon cell biochemistry and the supplementation type using PUFAs or SFAs, we have conducted experiments using CCD-18 Co cells and PA. Figure 9 shows the data obtained for CCD-18 Co cells upon the addition of PA.

One can see from Figure 9 that after the addition of PA (magenta (10 μM, 24 h) and violet (50 μM, 24 h) bars), Raman band intensity ratios calculated based on peaks typical for lipids (1304, 1444 cm^−1^) and proteins (1256, 1656 cm^−1^) for ER, LDs, and mitochondria typical for normal cells become more similar to the ratios typical for cancer cells, excluding dependences observed for the nucleus (which confirm the stability of the chemical composition of this organelle). This observation correlates well with the thesis of the adverse impact of PFAs on human cells.

Because the properties of human cells, membranes depend on the UFA/SFA ratio; Raman data analysis has also been performed for this organelle. Figure 10 presents the results of the studies.

Firstly, one can see from Figure 10 that FAs are effectively built into the cell membranes and, secondly, the strongest effect can be observed for LA.

To confirm the effect of the FA dose and FA type on vibrational properties of human colon cells, we performed a statistical analysis of the data and calculated the Pearson correlation coefficients for all analyzed samples. The Pearson coefficient represents the ratio between the covariance of two variables and their standard deviations and is essentially a normalized measurement of the covariance. Table 1 shows the results of the statistical analysis performed.

## 4. Materials and Methods

### 4.1. Cell Lines and Cell Culture

The CCD-18 Co cell line (ATCC^®^ CRL-1459™) was purchased from ATCC: The Global Bioresource Center (10801 University Blvd. Manassas, VA 20110, USA). The CCD-18 Co cell line was cultured using ATCC-formulated Eagle’s Minimum Essential Medium with L-glutamine (catalog No. 30-2003). To make the complete growth medium, fetal bovine serum was added to a final concentration of 10%. Every 2–3 days, a new medium was used. The cells obtained from the patient were normal myofibroblasts in the colon. The biological safety of the CCD-18 Co cell line has been classified by the American Biosafety Association (ABSA) as level 1 (BSL-1). The Caco-2 cell line was also purchased from ATCC and cultured according to the ATCC protocols. The Caco-2 cell line was obtained from a patient—a 72-year-old Caucasian male diagnosed with colon adenocarcinoma. The biological safety of the obtained material is classified as level 1 (BSL-1). To complete the medium, it was based on Eagle’s Minimum Essential Medium with L-glutamine, with the addition of fetal bovine serum to a final concentration of 20%. The medium was renewed once or twice a week.

### 4.2. Cultivation Conditions

The cell lines (CCD-18 Co and Caco-2) used in the experiments in this study were grown in flat-bottom culture flasks made of polystyrene with a cell growth surface of 75 cm^2^. Flasks containing cells were stored in an incubator providing the following environmental conditions: 37 °C, 5% CO_2_, and 95% air.

Cells used for research were seeded onto CaF_2_ windows (25 × 1 mm) at a low density of 10^4^ cells/cm^2^. After 24 h incubation on the CaF_2_, the standard growth medium was removed, and fatty acid solution diluted in medium in concentrations 10 μM and 50 μM was added for 24 h. After this time, the cells were rinsed with PBS (phosphate-buffered saline, Gibco, 10010023, pH 7.4 at 25 °C, 0.01 M) and then cells were fixed with formaldehyde (4% buffered formalin) for 10 min and washed once more with PBS. The Raman confocal measurements were made immediately after the fixation of the samples. All the fatty acid solutions used for the supplementation procedure in the investigation were prepared by diluting the compound in the pure culture medium.

### 4.3. Raman Imaging

All maps and Raman spectra presented and discussed in this paper were recorded using the confocal microscope Alpha 300 RSA+ (WITec, Ulm, Germany) equipped with an Olympus microscope integrated with an ultra-high-throughput spectrometer and a CCD camera. The average excitation power of the 532 nm excitation laser during the experiments was 10 mW (measured after the beam passed through the objective), with an integration time of 0.5 s for the low-frequency region. The laser was focused on the sample through a Nikon objective lens with magnification of 40×, intended for cell measurements performed via immersion in PBS. Spectral images were collected with a sampling density of 0.5 μm (the *z*-axis step size was equal to 1.5 μm). The obtained Raman spectra and all imaging data were analyzed using cluster analysis (CA), which was executed using the WITec Project Plus package (for the removal of cosmic rays and smoothing and background corrections). More details about the equipment, settings, and parameters can be found in previous works [66,67,86,87,88].

The normalization model, divided by norm (divide the spectrum by the dataset norm), was performed using Origin 2021 software according to the following formula:

$$V^{\prime }=\frac{V}{∥V∥}$$

$$∥V∥=\sqrt{{v_{1}^{2}+v}_{2}^{2}+\cdots v_{n}^{2}}$$

where $v_{n}$ is the *n*^th^ *V* value.

The normalization was performed for all Raman spectra presented in the manuscript. Origin software was also used to perform the ANOVA analysis, which was necessary to indicate statistically significant results (means comparison: Tukey model; significance level: 0.05).

### 4.4. Determination of the Appropriate Concentration of FAs Using the XTT Test

For each cell type, XTT ((2,3-Bis-(2-Methoxy-4-Nitro-5-Sulfophenyl)-2*H*-Tetrazolium-5-Carboxanilide) proliferation kit, catalogue number 20-300-1000, Biological Industries) tests were performed 24 h after the addition of FAs to the cells immersed in the culture medium. Preparation for the test included proper filling of the 96-well plate according to the procedure developed at the Institute of Applied Radiation Chemistry in Lodz. The wells were filled in such a way that each row contained a specific series of measurements. For example, in one row, all plates were filled with the medium; in another, the plates were filled with control samples containing only cells immersed in the medium; and in subsequent rows, plates were filled with cells in the medium with the addition of a specific concentration of FAs. Different concentrations of FAs were selected for the test:

1. For PA: 1 μM, 5 μM, 10 μM, 25 μM, 50 μM, and 100 μM.
2. For LA and EPA: 1 μM, 5 μM, 10 μM, 25 μM, 50 μM, and 100 μM.

After filling all of the 96-well plates, the samples were incubated at 37 °C. After 24 h from the addition of FAs, the XTT test was performed using Varioscan LUX Multimode Plate Reader from Thermo Fisher Scientific (Waltham, MA, USA). The measurement took about 3 h. After the completion of the study, the obtained results were analyzed using a spreadsheet, resulting in a bar graph showing the effect of the concentration of FAs on the survival of the tested cell type, taking into account the time since the addition of FAs.

Figure S2 shows the XTT viability tests conducted on human normal colon cells and human colon cancer cells supplemented with LA (Linoleic Acid, L1376, Merck Life Science Sp. z o. o, Warsaw, Poland), EPA (*cis*-5,8,11,14,17-eicosapentaenoic Acid, E2011, Merck Life Science Sp. z o. o, Warsaw, Poland), and PA (Palmitic Acid, (P0500), Merck Life Science Sp. z o. o, Warsaw, Poland). Please see Supplementary Materials for XTT viability test dates and a detailed description of the obtained results.

## 5. Conclusions

Using Raman imaging, we have proved that mapping mode can be effectively used to visualize single-cell substructures, which is helpful in the analysis of FA metabolic pathways. Using the cluster analysis algorithm, we have visualized the endoplasmic reticulum (ER), mitochondria, lipid droplets (LDs), and nucleus—the major organelles involved in the metabolism of FAs. We have analyzed the chemical composition of these organelles without and upon FA supplementation. Analysis of Raman band intensity ratios typical for lipids, proteins, and nucleic acids (I_1656_/I_1444_, I_1444_/I_1256_ I_1444_/I_750_, and I_1304_/I_1256_) proved that using Raman mapping, we can observe the metabolic pathways of FAs in various cellular compartments, including in ER, which is responsible for the uptake of exogenous FAs, de novo synthesis, elongation, and desaturation; in mitochondria, responsible for energy production; in LDs, specialized in fat storage; and in the nucleus, where fatty acids are transported via fatty-acid-binding proteins. These pathways serve as biomarkers of human colon cancerogenesis. Moreover, Raman studies of cell membrane composition showed the effective incorporation of FA molecules, with the strongest effect for LA. The spectroscopy studies were completed with XTT tests, which showed that the addition of LA or EPA for Caco-2 cells decreases their viability with a stronger effect observed for LA; the opposite effect was observed for PA. For normal cells, CCD-18 Co supplementation using LA or EPA stimulated cell growth, while PA had the opposite effect.

## Figures and Tables

**Table 1:** Pearson correlation coefficients calculated based on mean Raman spectra typical for normal human colon cells (CCD-18 Co) and cancer human colon cells (Caco-2) and cells upon FA supplementation. Data for EPA supplementation for 10 μM, 24 h, and 50 μM, 24 h; LA supplementation for 10 μM, 24 h, and 50 μM, 24 h; and PA supplementation for 10 μM, 24 h, and 50 μM, 24 h.

	*Pearson Correlation Coefficient*	*p-Value*
***Mean Raman spectrum of***	***CCD-18 Co cells***	
CCD-18 Co + EPA 10 μM, 24 h	0.98592	<0.05
CCD-18 Co + EPA 50 μM, 24 h	0.98073	<0.05
CCD-18 Co + LA 10 μM, 24 h	0.97283	<0.05
CCD-18 Co + LA 50 μM, 24 h	0.97381	<0.05
CCD-18 Co + PA 10 μM, 24 h	0.98524	<0.05
CCD-18 Co + PA 50 μM, 24 h	0.98133	<0.05
	***Caco-2 cells***	
Caco-2 + EPA 10 μM, 24 h	0.99138	<0.05
Caco-2 + EPA 50 μM, 24 h	0.95889	<0.05
Caco-2 + LA 10 μM, 24 h	0.96872	<0.05
Caco-2 + LA 50 μM, 24 h	0.96572	<0.05
CCD-18 Co + PA 10 μM, 24 h	0.96862	<0.05
CCD-18 Co + PA 50 μM, 24 h	0.98119	<0.05

## Data Availability

The raw data underlying the results presented in the study are available on request. Requests for access to those data should be addressed to the Head of Laboratory of Laser Molecular Spectroscopy, Institute of Applied Radiation Chemistry, Lodz University of Technology. Data requests may be sent by email to the Secretary of the Institute of Applied Radiation Chemistry: w3i34@adm.p.lodz.pl.

## Supplementary Materials

The following supporting information can be downloaded at: https://www.mdpi.com/article/10.3390/ijms25084508/s1.
