# Supplementary material for: Raman Imaging—A Valuable Tool for Tracking Fatty Acid Metabolism—Normal and Cancer Human Colon Single-Cell Study
Source: Int J Mol Sci. 2024 Apr 19;25(8):4508. doi: 10.3390/ijms25084508 (PMC11050638; doi:10.3390/ijms25084508)
Supplement: Supplementary file 1 [file ijms-25-04508-s001.zip › ijms-2961660-supplementary.pdf]

Supplementary Materials

Raman imaging - a valuable tool to track fatty acids metabolism - normal and cancer human colon single cell study

K. Beton-Mysur[1], M. Kopec[1], B. Brozek-Pluska[1]

[1]Lodz University of Technology, Faculty of Chemistry, Institute of Applied Radiation Chemistry, Laboratory of Laser Molecular Spectroscopy, Wroblewskiego 15, 93-590 Lodz and Poland

Some FAs can be synthesized in a human body. FAs biosynthesis is related to the carboxylation of acetyl-CoA, which leads to the formation of malonyl-CoA. This reaction is catalyzed by acetyl-CoA carboxylase, an enzyme containing biotin as a prosthetic group. [11] In this step of metabolism CO_2 is attached to acetyl-CoA to produce malonyl-CoA. ATP provides energy input. The same CO_2 is removed when the malonyl group condenses with the growing acyl chain. Acetyl-CoA and malonyl-CoA are then converted to their ACP derivatives. Acetyl-ACP and malonyl-ACP then condensed to form acetoacetyl-ACP using the enzyme 3-ketoacyl synthase. This reaction is driven by the release of CO_2 from the activated malonyl unit. Then, reduction processes take place (reduction of acetoacetyl-ACP to D-3-hydroxybutyryl-ACP, in which the reducing agent is NADPH, and the catalyst is β-ketoacyl-ACP reductase), dehydration (dehydration of D-3-hydroxybutyryl-ACP to crotonyl-ACP , in which the catalyst is 3-hydroxyacyl-ACP dehydratase) and subsequent reduction (reduction of crotonyl-ACP to butyryl-ACP, in which the reducer is NADPH and the catalyst is enoyl-ACP reductase). The butyryl-ACP obtained in this way enters the elongation cycle, which begins with the addition of a two-carbon unit from another malonyl-CoA molecule. Seven cycles of elongation ultimately lead to the formation of palmitoyl-ACP. This compound hydrolyzes to palmitate and ACP. The enzymes involved in catalyzing the synthesis of FAs form an enzyme system called fatty acid synthase (FAS). Palmitate synthesis requires: 8 molecules of acetyl-CoA, 14 NADPH, 7 ATP and 7 HCO_3^-, which plays a catalytic role.

The activated FAs (FA-CoA) may serve as substrates for the synthesis of glycerol esters (TGs) and phospholipids (PHLs) or can be transported to mitochondria whereby they undergo β-oxidation.

Generally, in a human body, FAs serve as structural or energetic compounds (a part is stored in lipid droplets, (LDs)), but the role of FAs and their metabolism pathways depend a.o. on their acyl chain length and the degree of unsaturation. The chain length may influence the rigidity of membranes built by FAs and the saturation determines the susceptibility of unsaturated FAs to oxidation. PUFAs are generally more prone to oxidation due to the presence of multiple double bonds. [13] Position of the double bond within the PUFAs molecules is also crucial from the functional point of view, since ω-6 PUFAs metabolites are generally proinflammatory, whereas ω-3 PUFAs act as anti-inflammatory compounds. [14]

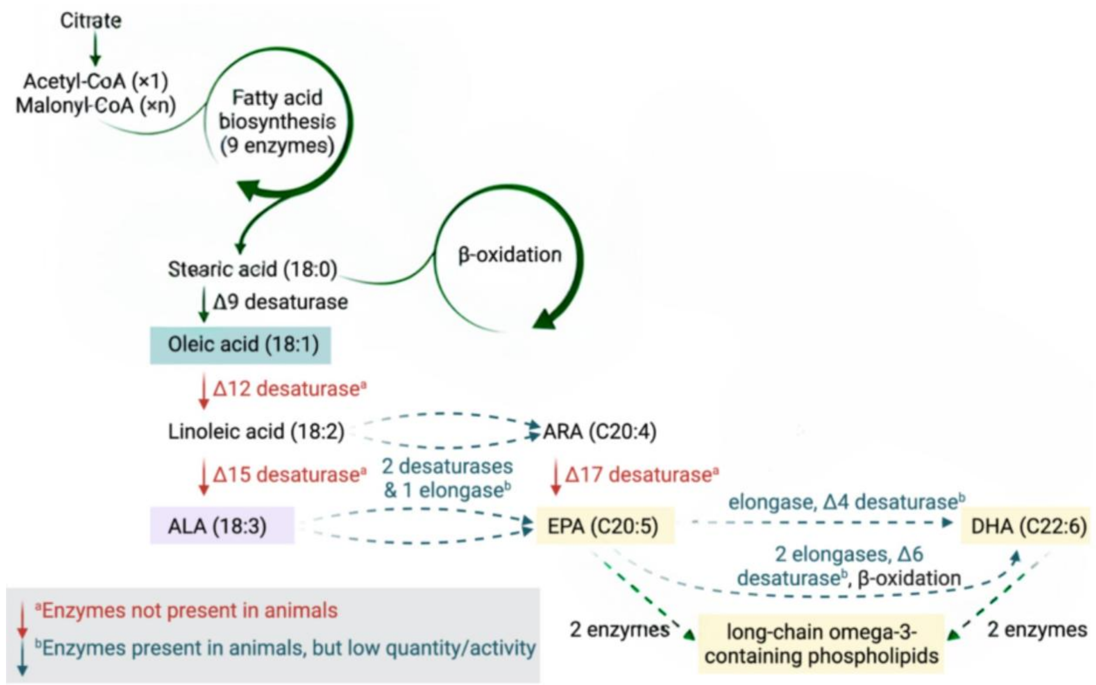

Figure S1. *Schematic presentation of reactions cascade leading to the synthesis of fatty acids (FAs) biosynthesis.*

Figure S2 shows the XTT viability tests conducted on human normal colon cells and human colon cancer cells supplemented with LA, EPA and PA.

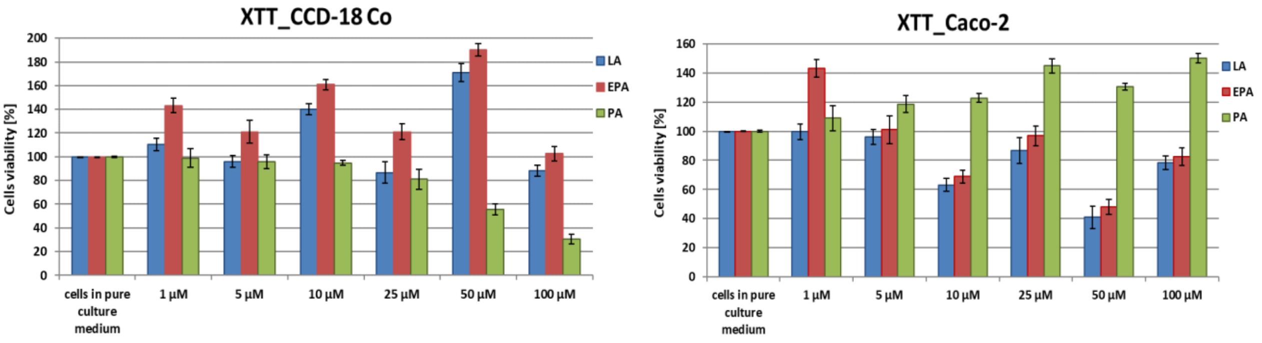

Figure S2: *XTT viability tests for human colon normal (CCD-18 Co) and cancer (Caco-2) cells upon FAs supplementation: EPA, LA, PA for different concentrations after 24h incubation.*

From the results obtained in XTT test it can be seen that the addition of LA or EPA for normal human colon cells stimulate cells for growing. Whereas addition of LA or EPA for cancer human colon cells decreases the viability of these cell type with a stronger effect observed for LA. The same tendency of presented data confirms the toxicity of FAs for overconsumption. Based on the cell viability test, it can be concluded that the addition of PA, which is a representative of SFAs, causes a significant reduction in the viability of healthy cells of the human colon. The opposite effect is visible for cancer cells, because the addition of PA causes faster cell growth, and increased survivalence, as shown in the bars above. PA results for both healthy and cancer cells are concentration dependent. The XTT test showed that the addition of UFAs is more beneficial for both healthy and cancer cells. The study of cell survival after the addition of FAs showed that FAs belonging to the ω-3 series can inhibit the development

of cancer tumors and reduce the risk of developing some types of CRCs. In terms of the anticancer effect of ω-3 fatty acids, it has been shown that EPA may inhibit the growth of cancer cells. The XTT study also showed stimulation of apoptosis processes and reduction of inflammation accompanying oncological diseases.

Below we present a single-cells analysis to identify FAs impact on human colon cells. To properly address biochemical changes, with the main focus on FAs metabolism in human normal and cancer colon cells upon FAs supplementation in comparison to not supplemented types, we investigated systematically how the Raman method responds to *in vitro* samples. Similarily, like for cells with EPA supplementation, we used Raman spectroscopy to record vibrational spectra and Raman maps at the single-cell level for human colon cell lines: CCD-18 Co (normal) and Caco-2 (cancer) over the molecular spectral range of 500–1800 cm^{-1} (the fingerprint region).
Data for LA supplementation are presented in Figures S3 (for CCD-18 Co line) and S4 (for Caco-2 line), for PA in Figure S5 (for CCD-18 Co line).

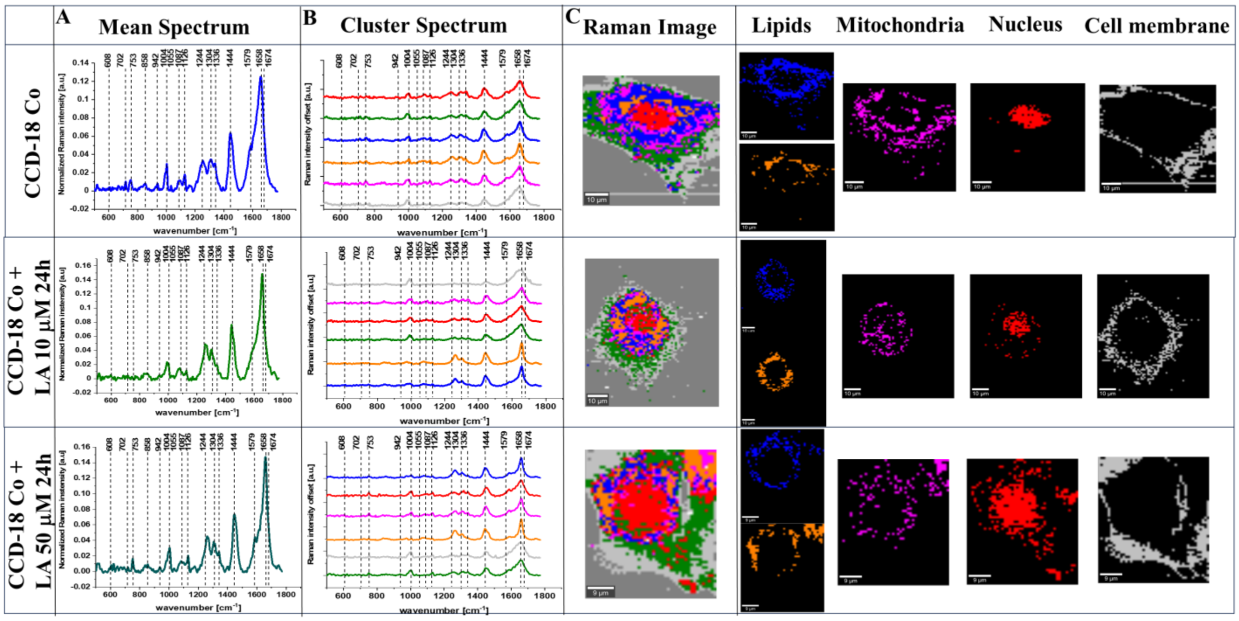

Figure S3. *The mean Raman spectra for cells as a whole (A), the mean Raman spectra typical for all clusters identified by using the Cluster analysis (CA) method (B), Raman images constructed based on CA method (C), and the single clusters from which: blue and orange corresponds to lipid rich regions , magenta corresponds to mitochondria, red corresponds to nucleus and gray corresponds to cell membrane identified by using CA method for human normal colon cells CCD-18 Co without any supplementation and upon LA supplementation for 24 h, for 10 μM and 50 μM. All cells were measured in PBS. The scale bar represents 10 μm. Colors of the spectra correspond to the colors of clusters.*

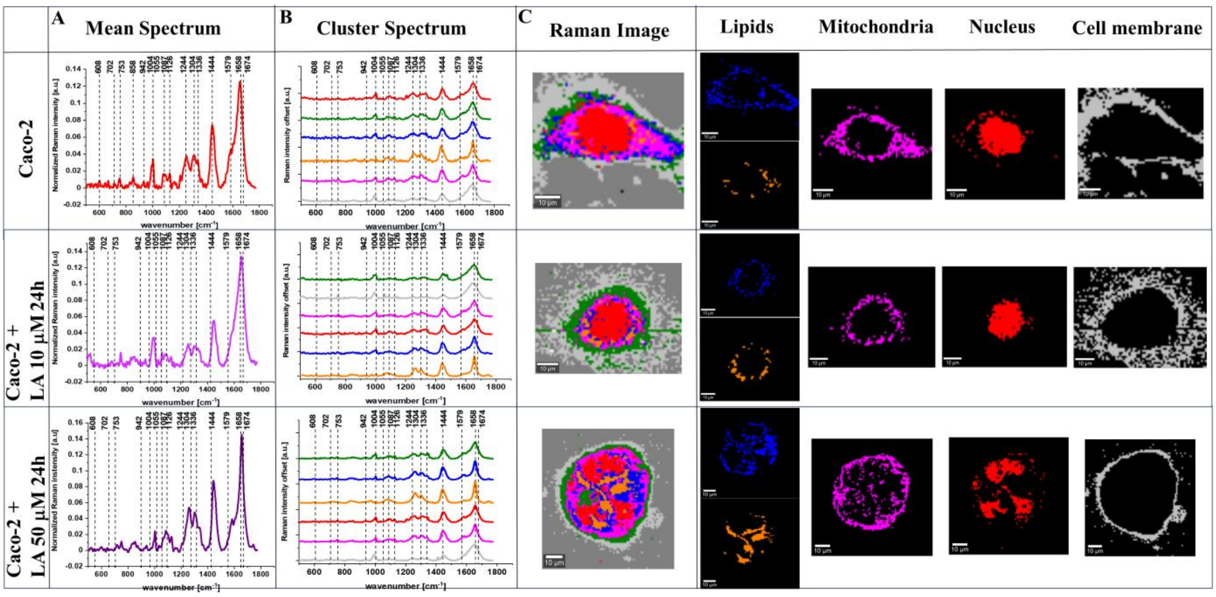

Figure S4. *The mean Raman spectra for cells as a whole (A), the mean Raman spectra typical for all clusters identified by using the Cluster analysis (CA) method (B), Raman images constructed based on CA method (C), and the single clusters from which: blue and orange corresponds to lipid rich regions, magenta corresponds to mitochondria, red corresponds to nucleus and gray corresponds to cell membrane identified by using CA method for human colon cancer cells Caco-2 without any supplementation and upon LA supplementation for 24 h, for 10 μM and 50 μM. All cells were measured in PBS. The scale bar represents 10 μm. Colors of the spectra correspond to the colors of clusters.*

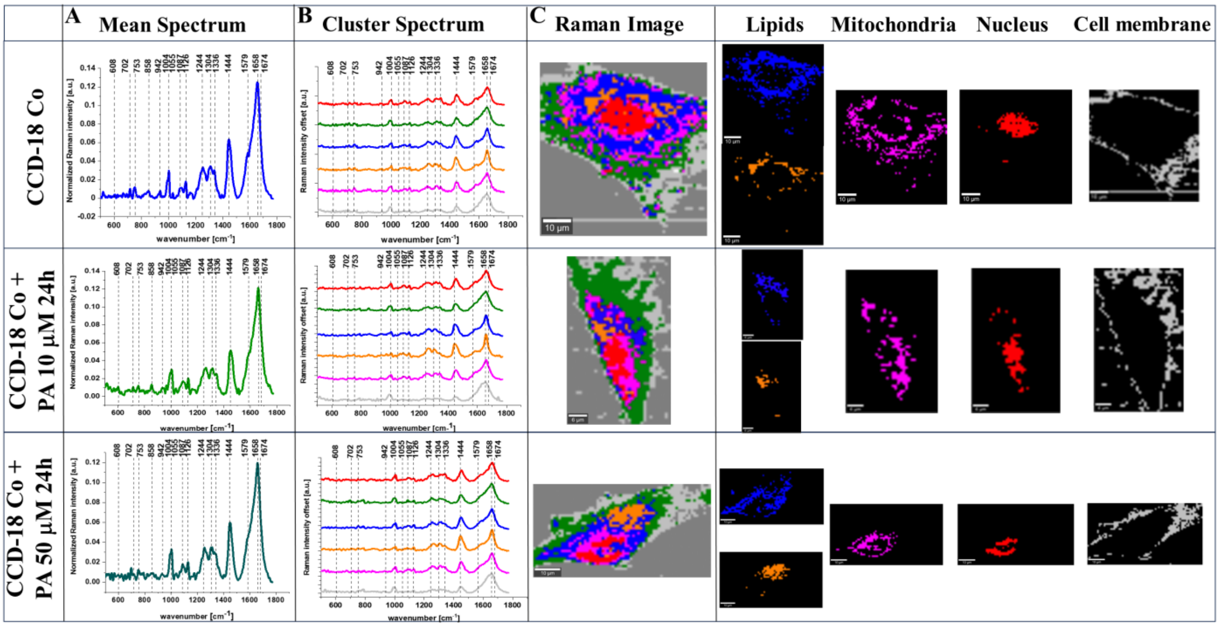

Figure S5. *The mean Raman spectra for cells as a whole (A), the mean Raman spectra typical for all clusters identified by using the Cluster analysis (CA) method (B), Raman images constructed based on CA method (C), and the single clusters from which: blue and orange corresponds to lipid rich regions, magenta corresponds to mitochondria, red corresponds to nucleus and gray corresponds to cell membrane identified by using CA method for human normal colon cells CCD-18 Co without any supplementation and upon PA supplementation for 24 h, for 10 μM and 50 μM. All cells were measured in PBS. The scale bar represents 10 μm. Colors of the spectra correspond to the colors of clusters.*

Scheme S1 shows the simplified illustration of RI experiments idea and the comparison of RI and fluorescence data for one cell chosen from our database including schematic diagram of using Raman imaging to track fatty acid metabolism.

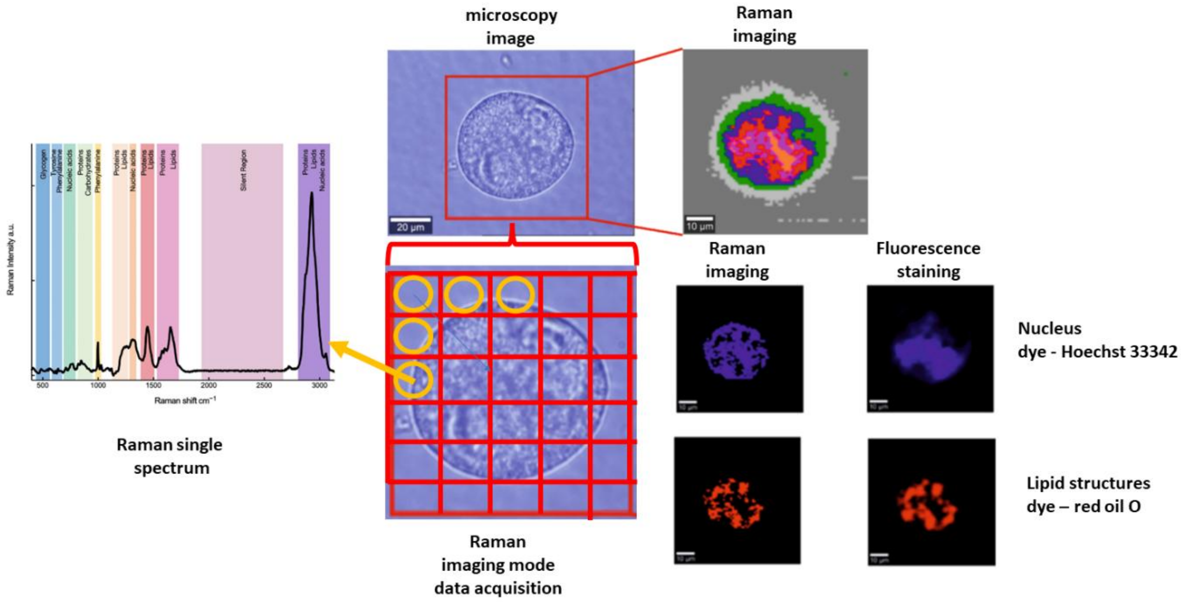

Scheme S1. *Schematic representation of the RI measurements idea and the comparison between RI and fluorescence staining data including schematic diagram of using Raman imaging to track fatty acid metabolism. Partially based on ref [70].*
